# Supplementary material for: Moral Distress in Healthcare Providers Who Take Care of Critical Pediatric Patients throughout Italy—Cultural Adaptation and Validation of the Italian Pediatric Instrument
Source: Int J Environ Res Public Health. 2022 Mar 24;19(7):3880. doi: 10.3390/ijerph19073880 (PMC8997869; doi:10.3390/ijerph19073880)
Supplement: Supplementary file 1 [file ijerph-19-03880-s001.zip › ijerph-1604061-supplementary.pdf]

Table S1. ***Italian Pediatric MDS-R*** **- Cognitive Interview.** Main highlighted problems and their solutions.

Italian Pediatric MDS-R - Cognitive Interview (n=14)	
**Heading**	"*Anche se non hai mai provato quella specifica situazione, indica comunque quanto spiacevole potrebbe essere nel caso essa si verificasse nella tua pratica clinica"-"Even if you have not experienced a situation, please indicate how disturbed you would be if it occurred in your practice".*
**Problem**	Although the interviewees found the indications being very clear, many respondents had difficulty assigning an intensity score to situations they had never experienced.
**Solution**	As an attempt to reduce this problem, the following sentence was added to the heading of the "intensity section" for each item:*"A prescindere che tu l'abbia vissuta o meno, descrivi quanto è, o immagina quanto potrebbe essere spiacevole per te vivere questa situazione in una scala compresa tra…" -"Regardless of whether you have lived it or not, describe how much it is, or imagine how disturbing it might be for you to experience this situation. Rate it on a scale between…".*
**Item 3**	*"Avviare azioni salva vita quando ritengo che possano soltanto prolungare l'agonia del bambino"-"Initiate life-saving actions when I think they only prolong the child's agony"*
**Problem**	The term *"prolungare l'agonia"-"prolong the agony"* is presents in the adult version and derived from the English *"only prolong death"*. However, in the Italian language, it also means "suffering" and we noticed that this often influenced the results of both frequency and intensity scores. Moreover, 4 respondents specified that they did not found ethically correct to tolerate the suffering of a dying patient into a critical care department, since sedation should be used in these events.
**Solution**	The term was then changed in "…*prolungare il processo irreversibile verso la morte"-"…prolong child's irreversible death".*
**Item 4**	*"Assecondare il desiderio dei genitori di non discutere della morte imminente con un bambino terminale che ne fa richiesta"-"Follow the family's request not to discuss death with a dying child who asks about dying".*
**Problem**	All the interviewees (14) initially had some difficulties in identifying the situation described. Four misunderstood that it was the parents who did not want to receive updates on their child's impending death. Fifty percent of them gave a 0 score for frequency, of which 6 had correctly understood the question. During the cognitive interview, many participants stated that, in their opinion, some comprehension difficulties arose because they generally care for sedated and mechanically ventilated end-stage patients who cannot speak verbally. However, 12 out of 14 were able to give a valid example regarding the situation expressed in the question, often tracing it back to end-stage cancer patients who were admitted in ICU for post-operative monitoring or with a worsening of their underlying disease, who ask about their medical condition.
**Solution**	Starting from interview 12, an option B of the question was inserted and respondents were asked to choose the clearer option. The version B was chosen. The item has been then modified as follows:*"Assecondare la richiesta dei genitori di non parlare della morte imminente con un bambino terminale che invece ti chiede della sua morte." - "Follow parents' request not to talk about imminent death with a terminal child who instead asks you about his death".*
**Item 9**	"*Aumentare per un bambino incosciente la dose di farmaci sedativi/oppiacei a un livello che ritengo possa accelerare il decesso" - "Increase the dose of sedative / opioid drugs for an unconscious child to a level that I believe will accelerate death".*

Italian Pediatric MDS-R - Cognitive Interview (n=14)	
**Problem**	Despite the use of the verb *“accelerate”*, 6 of the 14 respondents (43%) had some difficulty in interpreting the question and had, at least initially, intended to "cause" death, euthanasia, which means an action not permitted by the current Italian legislation. Many of them claimed that the correct term would be "accompany" and not "accelerate". Two had a long latency in responding, 3 changed their initial response, 2 asked for help. This conducts were more evident in 5 of the interviewees (36%) and were read as strategies implemented to manage the emotional conflict caused by the question. Ten out of 14 (71%) then understood, during probing-induced reflection, that the situation refers to the "end of life" of a dying patient. In 5 cases (36%) a strong emotional component arose, in two of them it was explicitly due to the fact that they did not understand that it was an "end of life" situation.
**Solution**	Given the Italian law on the end-of-life (*Legge n. 219/2017*), which allows an increase in sedatives/opiates to achieve the patient's comfort and in order to clarify that the question does not ask for something related to an illegal action for the Italian culture, it is decided to add the adjective *“terminale”-“terminal"* to the question which then resulted as follows:*“Aumentare per un bambino terminale incosciente la dose di farmaci sedativi/oppiacei a un livello che ritengo possa accelerare il decesso”-“Increase the dose of sedative/opioid drugs for an unconscious terminal child to a level that I believe will accelerate death”.*
**Item 11**	*“Assecondare i desideri dei genitori riguardo all'assistenza del bambino, anche se non sono d'accordo, per timore di un’azione legale”-“Follow the family’s wishes for the child’s care when I do not agree with them, but do so because of fears of a lawsuit”.*
**Problem**	Some of the interviewees suggested that by moving the preposition *“per timore di un’azione legale”-“for fear of lawsuit"* to the beginning of the sentence, it was easier for them to focus their response to the specific situation. Furthermore, it was also believed that it had been previously inserted at the end of the sentence only due to a literal translation from English.
**Solution**	It is therefore changed to *“Per timore di un’azione legale, assecondare i desideri dei genitori riguardo all'assistenza del bambino, anche se non sono d’accordo”-“For fear of lawsuit, follow the family’s wishes for the child’s care when I do not agree with them”.*

Table S2. Correlations between intensity and frequency scores. Both the Pearson index and the polychoric correlation index resulted in consistently low values (r < 0.3) thus demonstrating that the responses on Intensity and Frequency sub-scales of the *Italian Pediatric MDS-R* were independent.

Item	Pearson Correlation	Polychoric Correlation
1. Witness healthcare providers giving "false hope" to parents.	0.181	0.199
2. Follow the family's wishes to continue life support even though I believe that it is not in the best interest of the child.	0.113	0.161
3. Initiate extensive life-saving actions when I think that they only prolong death.	0.177	0.218
4. Follow the family's request not to discuss death with a dying child who asks about dying.	0.149	0.159
5. Feel pressure from others to order what I consider to be unnecessary tests and treatments.	0.221	0.262
6. Continue to participate in care for a hopelessly ill child who is being sustained on a ventilator, when no one will make a decision to withdraw support.	0.110	0.142
7. Avoid taking action when I learn that a physician or nurse colleague has made a medical error and does not report it.	0.028	-0.06
8. Work with a physician or a nurse who in my opinion is providing incompetent care.	0.095	0.079
9. Increase the dose of sedatives/opiates for an unconscious child that I believe could hasten the child's death.	-0.202	-0.253
10. Take no action about an observed ethical issue because the involved staff members or someone in a position of authority requested that I do nothing.	0.096	0.099
11. Follow the family's wishes for the child's care when I do not agree with them, but do so because of fears of a lawsuit.	0.111	0.075
12. Watch patient care suffer because of a lack of provider continuity.	0.244	0.267
13. Witness diminished patient care quality due to poor team communication.	0.228	0.253
14. Ignore situations in which parents have not been given adequate information to insure informed consent.	-0.018	-0.078

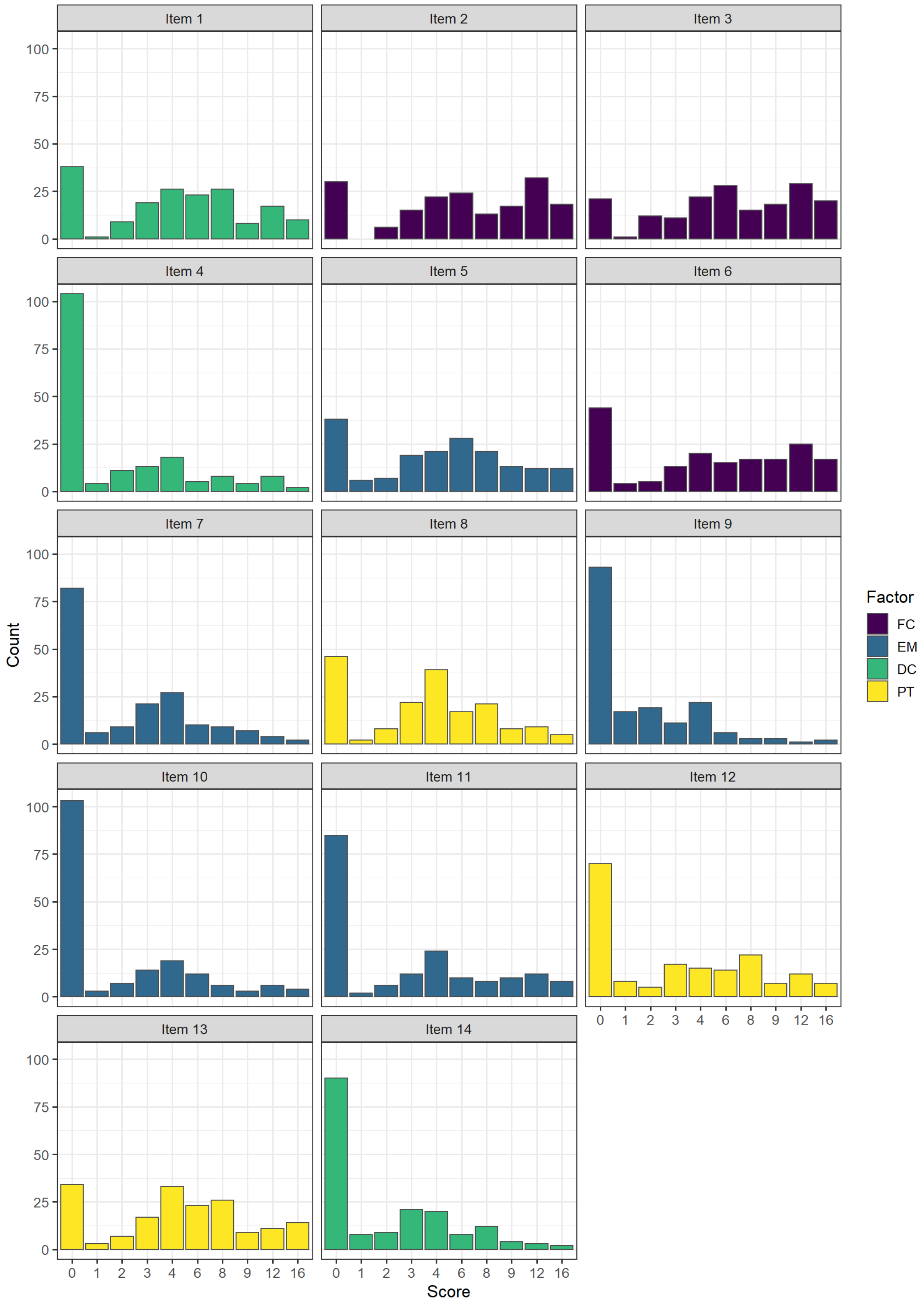

Figure S1 Graphical representation of the total scores obtained at the *Italian Pediatric MDS-R*. The number of subjects who obtain a certain score is shown on the vertical axis of each panel; the bars are colored according to the factor to which the item refers. The distributive forms are very similar to what we expected a priori, with a peak on zero which in some cases is very high. The distributions present heavy asymmetries due to a high frequency on zero.

Table S3. Correlations between items of the *Italian Pediatric MDS-R.* The matrix shows under the main diagonal the correlations between items, while reports the variance-covariance matrix on the rest of the cells.

Italian Pediatric MDS-R Cor-Var-Cov-3

Item	1	2	3	4	5	6	7	8	9	10	11	12	13	14
1	20.134	10.597	11.325	6.063	9.118	11.127	4.486	4.080	1.179	6.137	8.478	4.483	4.383	1.912
2	0.471	25.105	15.437	6.789	9.760	14.833	5.010	3.155	1.974	4.719	8.517	3.938	5.248	1.287
3	0.517	0.631	23.838	6.910	12.368	14.829	4.918	4.104	2.202	4.574	11.578	2.625	5.876	1.160
4	0.378	0.379	0.396	12.793	7.289	8.784	3.984	3.077	3.005	4.842	7.850	2.722	3.087	1.951
5	0.446	0.428	0.556	0.448	20.722	13.068	6.917	7.706	1.655	8.372	10.744	4.744	7.500	2.857
6	0.476	0.568	0.583	0.471	0.551	27.155	5.821	3.144	3.630	7.353	11.014	4.869	7.056	1.575
7	0.296	0.296	0.298	0.330	0.450	0.331	11.398	5.428	1.590	5.159	7.078	5.540	6.754	3.101
8	0.233	0.161	0.215	0.220	0.433	0.154	0.412	15.263	1.014	4.924	5.759	7.807	7.725	4.159
9	0.096	0.144	0.165	0.307	0.133	0.254	0.172	0.095	7.492	3.040	2.027	0.267	3.483	0.146
10	0.369	0.254	0.253	0.365	0.496	0.380	0.412	0.340	0.299	13.755	6.895	4.443	5.424	2.865
11	0.411	0.370	0.516	0.477	0.513	0.460	0.456	0.321	0.161	0.404	21.133	5.523	7.364	3.239
12	0.221	0.174	0.119	0.169	0.231	0.207	0.363	0.443	0.022	0.265	0.266	20.386	10.767	4.676
13	0.215	0.230	0.265	0.190	0.362	0.298	0.440	0.435	0.280	0.321	0.352	0.524	20.692	5.450
14	0.130	0.078	0.072	0.166	0.191	0.092	0.280	0.324	0.016	0.235	0.215	0.316	0.365	10.765

Table S4. Fit indices of adapted models. The models with the best fit was *Modified-2 Lamiani et.al* in which item 14 also saturates the PT factor, as well as the DC factor and the *Three* model which is modified Lamiani's model, in which the DC scale was dissolved by assigning its three items to the other scales (item 1 to the FC, item 4 to the EM, item 14 to the PT). The latter was chosen as the best solution (CFI and TLI values close to 0.95 and in any case above the 0.9 threshold; RMSEA value < 0.8 and even close to 0.6; SRMR value < 0.8; WRMR value just over 1). This model showed indeed the lowest AIC and BIC values, good indexes of adaptation, structurally simplicity without including two-factorial items.

**Model**	**AIC**	**BIC**	**CFI**	**TLI**	**RMSEA**	**SRMR**	**WRMR**
Unidimensional	6402.860	6491.793	0.7885242	0.7500741	0.11373450	0.09180723	1.553179
Lamiani et al.	6306.910	6414.9	0.9108165	0.8856943	0.07691666	0.06982528	1.176551
Modified-1 Lamiani et al.	6280.026	6388.015	0.9430654	0.9270275	0.06145628	0.05331164	1.062181
Three factors	6278.625	6377.086	0.9411469	0.9276266	0.06120347	0.05701859	1.082604
Modified-2 Lamiani et al.	6281.049	6392.215	0.9430373	0.9259485	0.06190895	0.05243136	1.057794
